# Supplementary figures and images for: RNA Sequencing of Pooled Samples Effectively Identifies Differentially Expressed Genes
Source: Biology (Basel). 2023 Jun 2;12(6):812. doi: 10.3390/biology12060812 (PMC10295764; doi:10.3390/biology12060812)

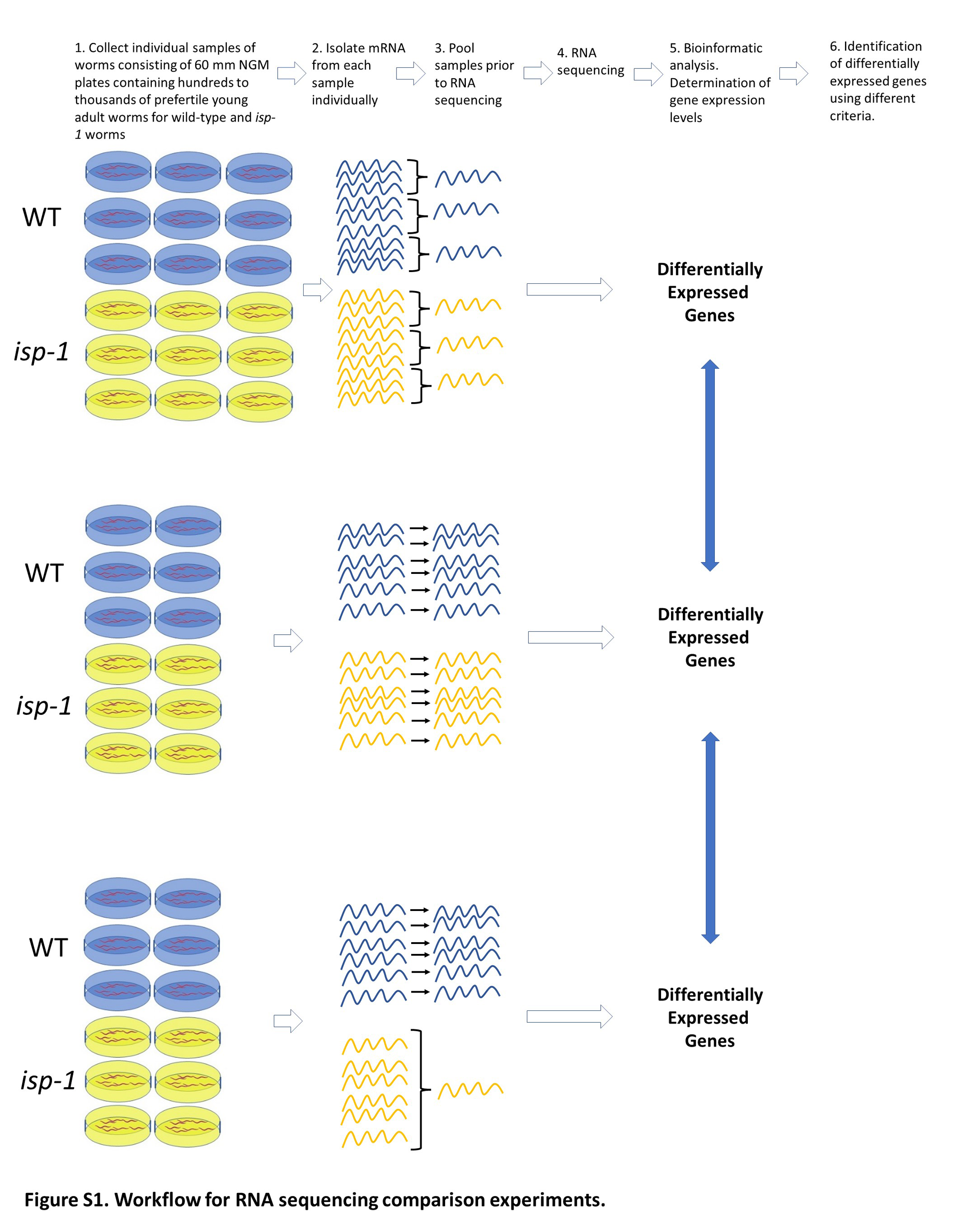

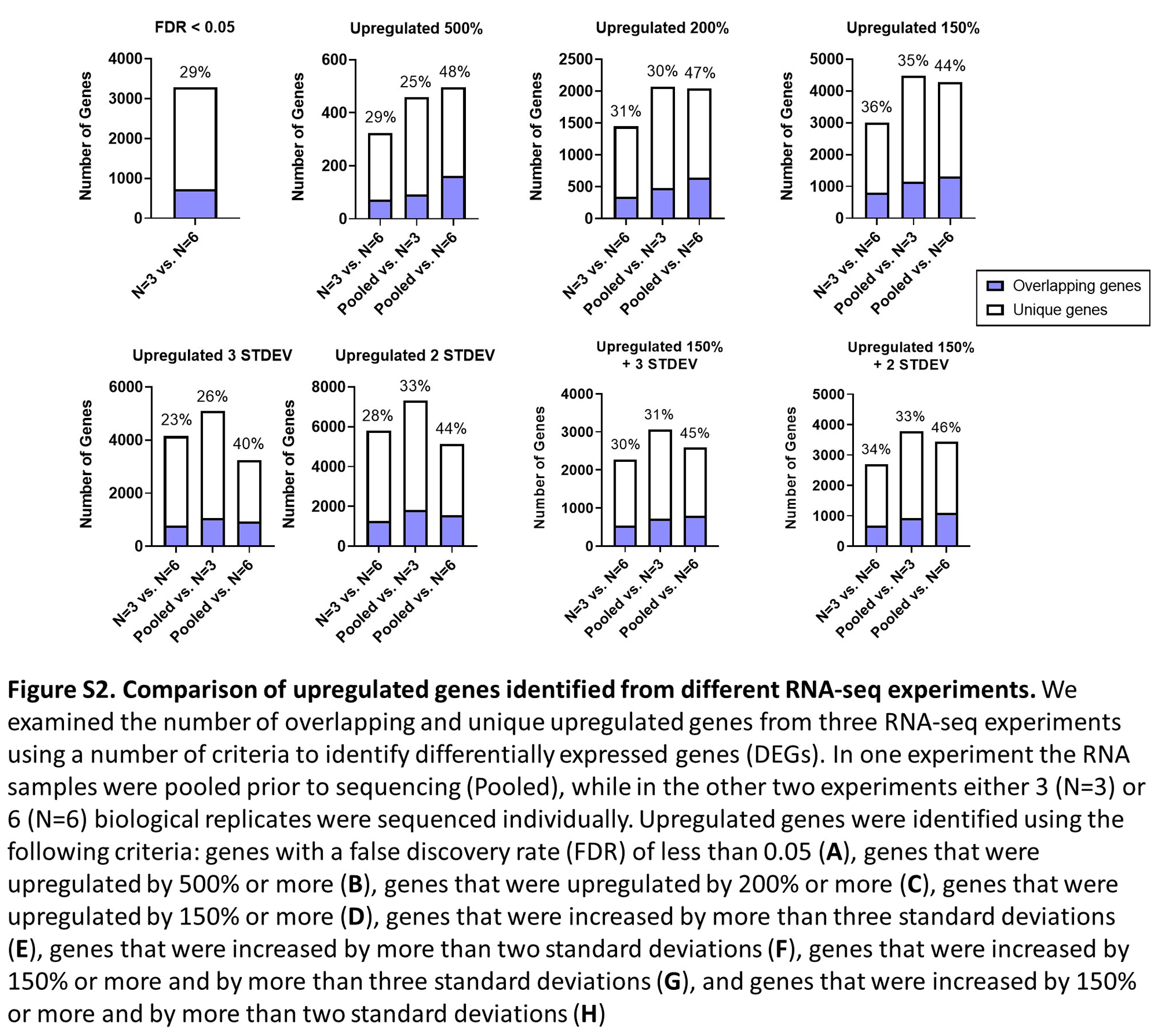

Supplement: Supplementary file 1 [file biology-12-00812-s001.zip › biology-2423290-supplementary.docx]
